# Sensing form - finger gaiting as key to tactile object exploration - a data glove analysis of a prototypical daily task

**DOI:** 10.1186/s12984-020-00755-6

**Published:** 2020-10-08

**Authors:** Werner Krammer, John H. Missimer, Simon Habegger, Manuela Pastore-Wapp, Roland Wiest, Bruno J. Weder

**Affiliations:** 1 Support Center for Advanced Neuroimaging (SCAN), Department of Diagnostic and Interventional Neuroradiology, Inselspital, University Hospital Bern, Bern, Switzerland; 2 Department of Neurology, Kantonsspital St. Gallen, St. Gallen, Switzerland; 3 Paul Scherrer Institute, PSI, Laboratory of Biomolecular Research, Villigen, Switzerland

**Keywords:** Object in-hand manipulation, Precise handling, Finger gaiting, Finger synchrony, Time series, Principal component analysis, Graph analysis

## Abstract

**Background:**

Motor hand skill and associated dexterity is important for meeting the challenges of daily activity and an important resource post-stroke. In this context, the present study investigated the finger movements of right-handed subjects during tactile manipulation of a cuboid, a prototypical task underlying tactile exploration. During one motor act, the thumb and fingers of one hand surround the cuboid in a continuous and regular manner. While the object is moved by the guiding thumb, the opposed supporting fingers are replaced once they reach their joint limits by free fingers, a mechanism termed finger gaiting.

**Methods:**

For both hands of 22 subjects, we acquired the time series of consecutive manipulations of a cuboid at a frequency of 1 Hz, using a digital data glove consisting of 29 sensors. Using principle component analysis, we decomposed the short action into motor patterns related to successive manipulations of the cuboid. The components purport to represent changing grasp configurations involving the stabilizing fingers and guiding thumb. The temporal features of the components permits testing whether the distinct configurations occur at the frequency of 1 Hz, i.e. within the time window of 1 s, and, thus, taxonomic classification of the manipulation as finger gaiting.

**Results:**

The fraction of variance described by the principal components indicated that three components described the salient features of the single motor acts for each hand. Striking in the finger patterns was the prominent and varying roles of the MCP and PIP joints of the fingers, and the CMC joint of the thumb. An important aspect of the three components was their representation of distinct finger configurations within the same motor act. Principal component and graph theory analysis confirmed modular, functionally synchronous action of the involved joints. The computation of finger trajectories in one subject illustrated the workspace of the task, which differed for the right and left hands.

**Conclusion:**

In this task one complex motor act of 1 s duration could be described by three elementary and hierarchically ordered grasp configurations occurring at the prescribed frequency of 1 Hz. Therefore, these configurations represent finger gaiting, described until now only in artificial systems, as the principal mechanism underlying this prototypical task.

**Trial registration:**

clinicaltrials.gov, NCT02865642, registered 12 August 2016.

## Background

Motor hand skill is important for meeting the challenges of daily activity and its loss a critical consequence of stroke [1, 2]. The requisite manual dexterity relies on motor control exerted during active touch, which is essential to tactile object manipulation and exploration [3]. The relationship between tactile object manipulations and dexterity is evident in proposed definitions of the latter: “(The) process in manipulating an object from one grasp configuration to another” [4] or “(The) capability of changing the position and orientation of the manipulated object from a given reference configuration to a different one” [5]. Roland and Mortensen [6] developed a theoretical model of human somatosensory exploration of kinesthesia, macrogeometry, size and shape which describes the input-output relationships of tactile exploration. Using their fully quantified macrogeometrical stimuli, i.e. a set of parallelepipeds and spheres of identical volume representing non-real objects, we and others have verified in functional magnetic resonance imaging (fMRI) studies three modes of exploration in extended actions of digital object exploration [7, 8]. These modes consist of coordinated dynamic digital movements of the fingers: mainly thumb, index and middle finger, including intervals of rotating and encompassing the object with the three middle fingers. As measured by video-monitoring, the dynamic movement of the thumb consumed the most time [7, 9]. These modes of exploration disappeared in over-learned pure motor sequences at high frequency [10].

As done in previous fMRI studies [8, 11] assessing somatosensory discrimination, we investigated with the digital glove a task requiring the coordinated dynamic finger movements underlying tactile exploration in the absence of cognitive load. From a behavioral perspective, our paradigm consists of a series of changing elementary precision grasps, requiring multiple independently controlled contacts to optimize in-hand object orientation [12, 13]. According to Landsmeer such precise handling of the adapted fingers enables the subject to perform intrinsic hand movements without moving the arm [14, 15]. This sequence of manipulations has been described in artificial systems as finger gaiting; it requires multiple independently controlled contacts, designated virtual fingers, to optimize the object orientation during one motor act [12, 16, 17]. In gaiting, the set of constraining contacts is exchanged, where grasping fingers are replaced once they have reached joint limits by free fingers which continue the motion [13].

In the present study, twenty-two normal subjects manipulated with the digital glove repeatedly a cuboid at a frequency of 1 Hz, as instructed previously via a video. The time series of the 19 sensors observed to be associated with dynamic digital movements were subjected to a principal component analysis (PCA) for all subjects and sessions, yielding sensor and temporal patterns. The spatial patterns were classified using cluster analysis to establish commonality of the patterns. Graph and frequency analysis of individual finger movements yielded temporal aspects of the manipulation. The finger movements of a single selected subject in 3D space illustrated the manipulation. We propose that the short motor actions performed during the task can thus be decomposed into single motor acts of opposing thumb and finger configurations represented by the principal components. The decomposition thereby permits the characterization and quantification of the dynamical digital movements constituting the manipulations. This characterization is the precondition for classifying the task as finger gaiting in the taxonomy of within hand prehensile manipulation [16]. More importantly, the description of the patterns in healthy subjects provided by our study is intended to serve as a standard in monitoring post-stroke sensori-motor rehabilitation in patients with hand paresis and in the development of robotic tactile perception systems.

## Subjects and methods

### Subjects

Twenty-two healthy right-handed subjects, 10 males and 12 females ranging in age between 42 and 84 years, were included in the study. Their handedness scores according to the Edinburgh Handedness Questionnaire [17] ranged between 50 and 100. More detailed demographic data are shown in Table 1. The subjects had no prior history of psychological disorders, achieved normal Mini-Mental State Examination (MMSE) scores, and showed no abnormalities in MRI brain scans. The study received ethical approval from the Kantonale Ethikkommission Bern (KEK), 3010 Bern, Switzerland. Prior to the study all participants gave written informed consent before enrollment, according to the Declaration of Helsinki [18].

**Table 1:** Demographic subjects’ data

ID	gender	age (years)	LQ	MMSE
1	m	74	90	27
2	f	73	60	29
3	m	42	100	30
4	f	48	60	29
5	f	65	50	27
6	f	71	100	28
7	m	47	100	30
8	f	52	100	30
9	f	59	90	29
10	m	53	100	26
11	m	54	70	29
12	f	47	100	30
13	f	51	100	29
14	f	56	100	29
15	f	59	80	30
16	f	69	100	28
17	m	84	100	28
18	m	83	80	29
19	f	69	90	29
20	m	75	100	28
21	m	68	100	27
22	m	71	100	29
N or Median	10 m / 12 f	62	100	29
Range		42–84	50-100	26-30

(*m* male, *f* female, *LQ* laterality quotient, *MMSE* Mini-Mental State Examination)

### Sensori-motor assessment

Sensori-motor function was assessed with five measurements for both hands: (1) Power grip was measured using a Jamar hydraulic hand dynamometer [19]; (2) Precision grip was measured with a Jamar hydraulic pinch gauge [19]; (3) Motor hand skill was determined using one of the seven timed subtests comprising the Jebsen-Taylor Test (JTT) [20], namely, “Picking Small Objects” (PSO); (4) Two-point discrimination (2PD) was measured on the index finger tip using a graded caliper [2-point Discriminator, Medwork Instruments, Vancouver, Canada] [21]; and (5) tactile object recognition (TOR) was tested using a standardized protocol employing 30 everyday objects as previously described [22]. The assessments were intended solely to confirm normal sensori-motor abilities in the subjects; they were not incorporated in the following analyses.

### Data glove instrumentation and calibration

**Fig. 1:**
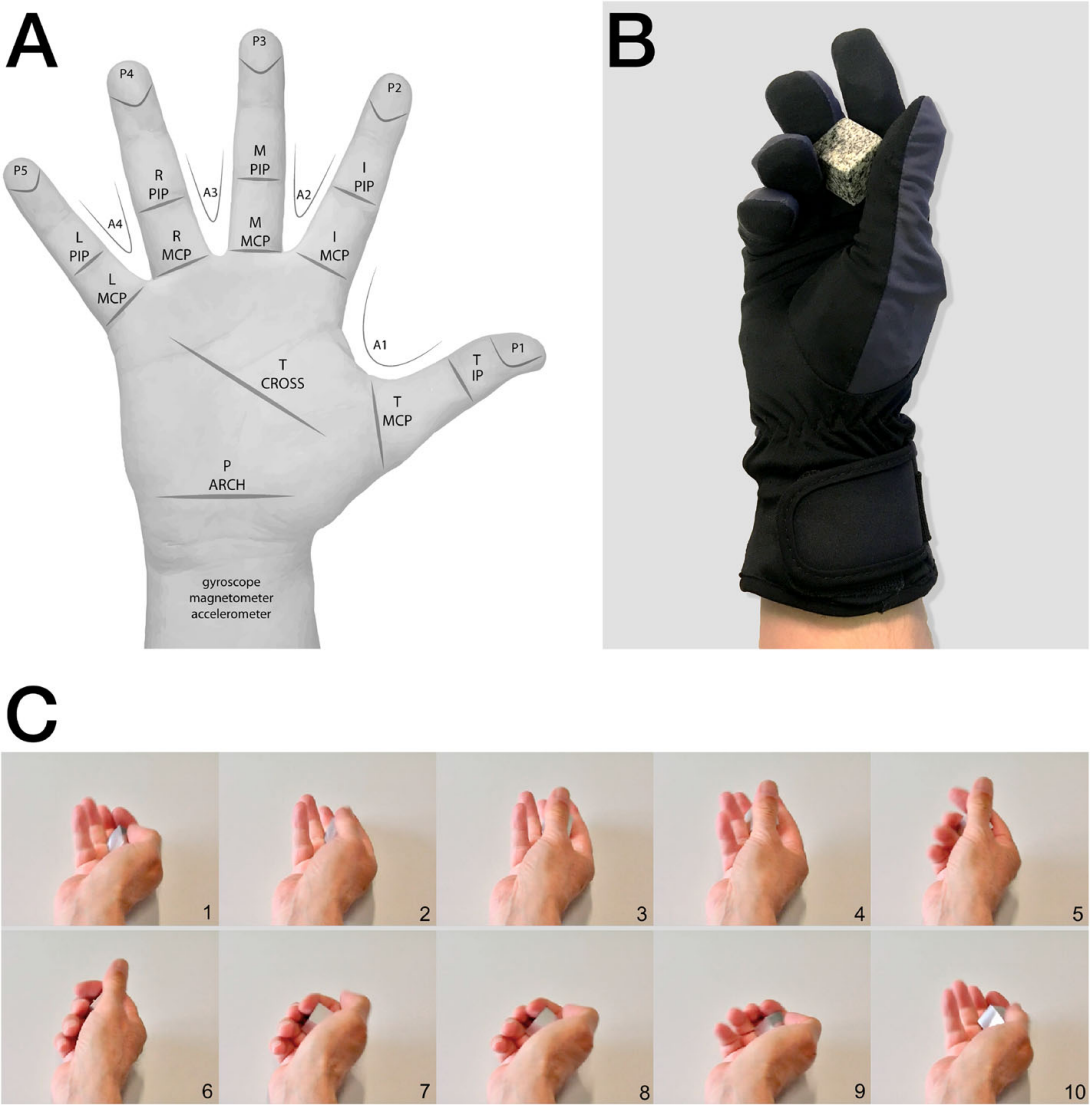
Labels and locations of sensors, data glove and consecutive steps of manipulation. **a** Labels of all sensors, **b** representation of a hand in the data glove holding the cuboid, **c** image sequence of instruction video showing manipulation of cuboid (one motor act)

We employed the VMG 30™ data glove from Virtual Motion Labs [Virtual Motion Labs, LLC., 3010 LBJ Freeway, Dallas, Texas 75,234 (see http://www.virtualmotionlabs.com)]. The glove is equipped with 29 sensors of which 16 are bend sensors. Two finger bend sensors per finger measure the movement extent in the metacarpo-phalangeal (MCP) and proximal interphalangeal (IP) joints, and two finger bend sensors at the thumb measure movement extent in the MCP and IP joints. Four sensors between the fingers measure abduction versus adduction. One palm arch sensor detects spatial configuration related to the proximal and distal transverse arch of the hand described by Hertling and Kessler [23]. One thumb cross sensor (Tcross) detects the complex movement of the thumb during finger opposition at carpo-metacarpal (CMC) joint. Five sensors situated at the finger tips measure pressure and eight sensors measure hand and wrist orientation (Fig. 1 a, b). Calibration of the data glove set as default consisted of seven calibration stages detailed in Supporting Information. Bend sensors were calibrated between values of 1000 and 0. The maximum occurred by flexion in the MCP and proximal interphalangeal (PIP) joints, adduction of fingers, transaxial extension of the thumb and forming the palm arch and the minimum value of 0 by maximal extension in the finger joints, abduction of fingers, resting position of the palm arch and CMC joint of thumb. Finger pressure sensors were calibrated between a value of 1000 for no pressure and 0 for maximum pressure.

### Task performance

The sensori-motor task to be performed by the subjects consisted of regular single motor acts at a frequency of 1 Hz in which the opposed thumb and fingers of one hand surround the cuboid in a continuous and regular action. Consecutive steps of the motor act as displayed in the instruction video are depicted in Fig. 1c, which shows successive phases of the thumb and finger trajectories. Each phase begins with a transaxial movement of the thumb versus the ring finger. During the concerted action of thumb and fingers, the thumb exerts tangential forces that produce a marked rotation of the object, anticlockwise in the right hand, and clockwise in the left. In the terminology of Bullock et al. [13], 1) the action is prehensile, 2) the stabilizing fingers change continuously during one motor act, 3) the cuboid moves, guided by the tip of the thumb, relative to the contact points of the virtual fingers (i.e. in this task index, middle and ring finger) [12], 4) thumb and fingers move relative to the reference frame defined by the hand base, and 5) the motor sequence of fingers and thumb is repeated at the given frequency.

The cuboid was made of granite with a density of 2.6 g/cm^3^ and side lengths of 2.254 × 2.254 × 2.257 cm resulting in a total volume of 11.5 cm^3^ and weight of 29.9 g, comparable with those of the aluminum cube used in. A video was filmed to instruct the subjects how to perform the task. This video consisted of three, 20 s long, consecutive segments: (1) fixation, (2) observation, (3) active manipulation, each announced by written instruction on a blank white screen for 4 s. “Fixation” showed a hand holding the cube, however, had only the function of rest pause to prevent fatigue and thus, keep concentration high; “Observation” showed the same hand manipulating the cuboid at the prescribed 1 Hz; and upon “Active manipulation” the subjects were given the cube by the study physician and requested to manipulate the cube at the required speed as shown in the video sequence displayed during the segment “Observation” on the screen. A right hand was shown for the right hand sensori-motor task and a left hand for the left hand sensori-motor task. The 3 segments were repeated six times showing 3 male and 3 female hands and resulting in a total video length of 7.2 min. In-house software recorded the sensor data only during the 20 s of active manipulation.

During task performance, subjects were seated at a desk on which was placed a computer screen with their hands supine on the desktop. To ensure that the subjects understood the motor task, it was explained by the study physician, and subjects requested to manipulate the cuboid with the left and right hand without the data glove for about 10 s as shown by the physician. Then the calibrated data glove was put on the non-dominant left hand of the subject and checked for fit by the physician. The video was started when the subject’s hand was relaxed on the table top. When the instruction “Active manipulation” appeared on the screen, the physician placed the cuboid in the subject’s hand; after completion of the segment, the cuboid was removed. The glove calibration procedure required a break of about 2 min between acquisitions with the left and right hand.

### Data sampling

Data were acquired with software programmed in house and based on the Software Development Kit provided by Virtual Motion Labs. Pre-study testing of the signals produced by the task indicated that they could be most efficiently encoded at a frequency of 50 Hz, implying time frames of 20 msec. This frequency appears sufficient as indicated by the published critical thresholds of about 20 Hz for steady visual perception and 10 Hz for visual parsing [24]. One action of consecutive manipulations, denoted a run, consisted of 1000 time frames. In order to i) exclude irregularities as the subject adjusted to the prescribed frequency observed in the instruction video and ii) to impose a standard for subsequent analysis, only the last 800 time frames, i.e. 16 s, of each run were analysed.

### Data analysis

**Fig. 2:**
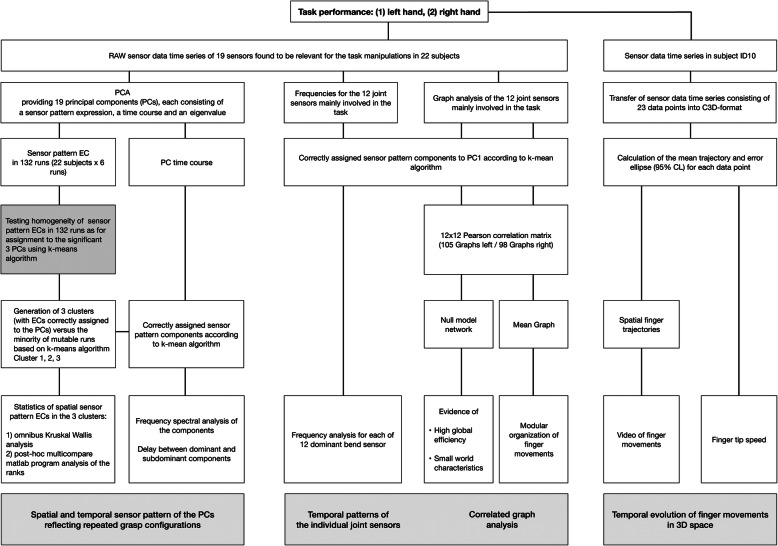
Comprehensive analysis of glove data. Note: Main topics outlined in the grey boxes are reviewed in separate paragraphs of discussion

A graphical representation of the analysis procedures is shown in Fig. 2.

The nineteen sensor time courses of each run and subject associated with prehensile in-hand manipulation were submitted to PCA (see Results). Separate analyses were performed for each hand. PCA was performed using in house software written in Matlab [The Mathworks, Inc., Natick, MA] based on the algorithm described by Alexander and Moeller [25]. The sensor amplitudes for each sensor in the 800 time frames were entered in a matrix. The rows corresponded to the 800 time frames and columns to the 19 relevant sensors of a run. PCA was applied to a residual matrix. Using the singular value decomposition implemented in Matlab, the residual matrix was then decomposed into 19 principal components (PC). Each PC consisted of a sensor expression pattern, a time course and an eigenvalue. The sensor expression coefficients (ECs) describe the amount each sensor contributes to the component. The time course represents the variation of the component with time and the eigenvalue characterizes the fraction of variance described by each component. The sensor ECs and time courses of a PC are orthonormal; the orthogonality reflects the lack of statistical correlation among the principal components.

Preliminary analysis showed that the first three PCs of each run and subject explained about 75% of the variance, a number consistent with the Guttman-Kaiser criteria (GK) for salient PCs [26]. Further analysis was therefore restricted to these first three PCs.

### Spatial sensor patterns

Statistical analysis of the sensor ECs must take into account the indeterminacy of the signs associated with multilinear models such as PCA [27], i.e. two different sets of coefficients expressing the same pattern might differ only in the signs of the sensor contributions. Before analysis of the subject cohort, alignment of the ECs was therefore necessary. Alignment was performed in two stages. First, pairwise correlations of the ECs were computed for the six runs of each subject and PC and the signs adjusted to yield the highest positive correlation. Second, the realigned ECs of the 22 subjects were submitted to a second pairwise correlation analysis to determine the most favorable alignment among subjects. Based on the two steps of the alignment procedure and preliminary analyses of the principal components, we then assigned a positive sign to reflect increased bending of the thumb cross, MCP and/or PIP finger sensors. Thus, sensors yielding prominent positive signals indicate bending movements or less pressure synchronous with the selected finger sensors. Sensors yielding prominent negative signals indicate that the bending or pressure are out of phase compared to sensors exhibiting a positive sign, but with the same time course.

In order to assure the homogeneity of the component ECs for the complete cohort, k-means clustering was applied to the 3 PCs in all 132 runs and subjects, i.e. 6 runs for the 22 subjects. An iterative method for partitioning data, k-mean clustering yields mutually exclusive clusters after determining their central members. Therefore, each EC is assigned to a cluster and its distance to the central member, denoted centroid, is computed. Homogeneity of the coefficients implies that the clusters should correspond to the rank of the PC in explaining the variance of the coefficients, i.e. the PCs explaining the greatest variance would compose one cluster, the PCs explaining the second greatest variance a second cluster, and so on. To be consistent with the number of PCs considered in each run, we limited the number of clusters to three. We implemented the clustering using the program k-mean of Matlab. The distance between centroid and cluster member was computed using the option “correlation”, as suggested by the alignment procedure.

In order to evaluate the salience of the individual sensors in the task, medians, percentiles and confidence levels (CL) for the correctly identified component ECs were computed and compared with the centroid. Correctly identified coefficients are those for which the PC is labeled as belonging to its corresponding cluster, i.e. the dominant PC, PC1, of a particular run and subject is correctly identified if it is labeled as belonging to the cluster characterized by a predominance of PC1’s. To confirm the salience of individual sensors, a Kruskal-Wallis test of the sensor distributions, corrected for multiple comparisons of ranks, was performed using the Matlab programs, kruskalwallis and post-hoc multicompare.

### Temporal sensor patterns

To investigate the temporal properties of the PC clusters, frequency spectral analysis was applied to the time courses of correctly identified PCs. In addition, time delays between PCs for each run and subject were computed using the Matlab program finddelay. The sampling frequency of 50 Hz determined the maximum delay of 25 frames in the program, corresponding to one half of a sampling cycle. This procedure allows for assessing the hypothesis of finger gaiting underlying one complex motor act as posited, i.e. testing whether the distinct grasp configurations, as reflected by the PCs, occur at the same frequency of 1 Hz and, thus, in the same time window of 1 s.

In addition to the PCA, the frequencies and time delays among twelve individual sensors for all runs and subjects correctly assigned to Cluster 1 for both hands were analysed as represented by the central column of Fig. 2. The sensors comprised the ten finger bends (i.e. related to MCP and PIP joints, respectively IP joint of the thumb) and thumb cross (i.e. related to CMC joint and palm arch sensors). To reduce the noise in the time courses, the time courses were first filtered using a finite impulse response (FIR) filter with low pass cutoff frequency of 10 Hz. To achieve similar gain levels, they were normalized such that the magnitude of the maximum amplitude was unity. This preprocessing was implemented using the Signal Processing Toolbox of Matlab. The frequencies were determined by the time difference between signal maxima using the Matlab program findpeaks, Matlab. The time differences between minima and null positions confirmed the frequencies. The delays were limited to maximum delay of 25 frames as above.

### Graph analysis of selected sensor time series

Using the same time series of the 12 MCP/PIP (IP) finger bends, palm arch and thumb cross sensors included in the PCA and cluster analyses, we performed graph analysis with GraphVar (Release V2.01) [28] as implemented in Matlab. Restricting to runs for which PC1 was assigned to the associated cluster, the analysis required first calculation of the 12 × 12 Pearson correlation matrices for correctly assigned runs of each hand. From these were calculated mean matrices yielding a weighted undirected graph with 12 nodes and 66 edges for each hand. Negative weights, corresponding to negative correlations, were retained. To investigate subnetworks, the graphs were thresholded in steps of 0.05 for positive and negative weights. Global efficiencies for both graphs were calculated without thresholds. A null model network consisting of 100 random fully connected weighted graphs generated with 1000 iterations served as basis for comparison using the Mann-Whitney-U-Test. Finally, the graphs were submitted to the Louvain community detection algorithm [29] as implemented in the brain connectivity toolbox [30] using a gamma of one in order to determine the modularity of the graphs.

### Temporal evolution of finger movements in space

**Fig. 3:**
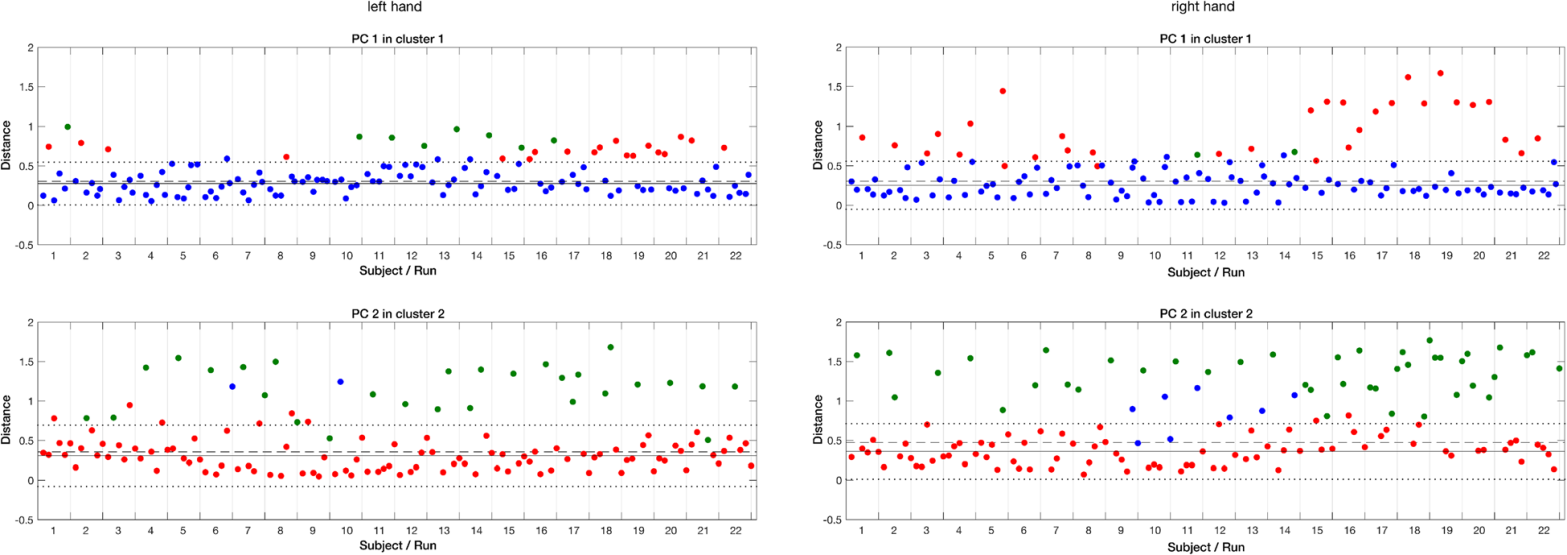
K-mean cluster classification of sensor patterns for PC1 and PC2 of left and right hands. The clusters are defined by the dominant PC, i.e. cluster 1 by PC1, cluster 2 by PC2 and cluster 3 by PC3 (Fig. S2, Supplemental Material). The distances are derived from the correlation between the cluster centroid and the spatial patterns of a run. The colour blue denotes the PC1’s, red the PC2’s and green the PC3’s. The medians, means, and 2 s bands of the dominant PC’s of a cluster are represented as dashed, solid and dotted lines, respectively. Misassigned runs are paled

To complement and illustrate the group PCA and temporal analysis of individual sensors, we acquired 3D data for male subject ID 10 in an additional acquisition. This analysis is represented on the right of Fig. 2. The group cluster analysis showed that PC1 of the subject had been assigned to the corresponding cluster in all runs of the right and in most runs of the left hand (Fig. 3, Fig. S2). Software provided by Virtual Realities converts the raw sensor data into the C3D file format (www.c3d.org) used in biomechanics, animations and gait analysis laboratories. This format comprises 23 data points representing a standardized 3D hand model, each consisting of x, y, and z values in millimeters. Because the finger tips play a central role in the task, we focused on the five data points representing the end of the distal phalanges to calculate spatial finger trajectories and average speed. A trajectory was defined as the points between consecutive maximal extensions of the thumb derived from repeated manipulations, as determined by the program findpeaks of Matlab.

As in group acquisitions, the data were acquired in 6 runs of 16 s each. However, the prescribed rate for the 3D acquisition mode was 36.97 Hz. Since the time between maximum extensions of the thumb varied, the number of trajectories was less than optimum: 80 for the right hand and 75 for the left. The speed of finger movement were then computed by dividing the path length of the trajectory by its duration. From the ensemble of trajectories for each hand were calculated a mean trajectory and 95% CL region, the latter using an error ellipsoid for each time point with the Matlab program error ellipse (https://ch.mathworks.com/matlabcentral/fileexchange/4705-error_ellipse). For visualization, the trajectories of approximately 37 frames were resampled to 100 frames and the mean trajectories and CL region displayed (Video 1) using the open source software Mokka version 0.6.2 (https://biomechanical-toolkit.github.io/mokka/).


**Additional file 1: Video 1.** Mean spatial trajectories of finger movements in 3D space. The video shows the mean spatial trajectories related to the tip of end phalanges as well as the vertex of the joint angles of finger and thumb sensors in individual ID10. The 3D dimensions of the trajectories correspond exactly to those of the workspace in Fig. 6, which is related there to the thumb and finger tips. The video displays the succession of 10 manipulations in normal and three in slow motion. Note: The 3D-Model of the left and right hand are in different space related to the preferred subject’s hand position on the desktop, however the x, y and z-axis have the same aspect ratios.

## Results

### Sensori-motor assessment

As indicated in Table S1 in Supporting Information, the results of the sensori-motor assessments were consistent with published data regarding age- and gender-matched healthy controls for power and precision grip [19], PSO [20], two point discrimination and TOR [22].

### Spatial sensor pattern

An initial PCA of all time series of all 29 glove sensors for both hands of all subjects showed that 10 sensors, including the 8 sensors comprising the hand and wrist quaternion and the pressures sensors P4 and P5 yielded ECs significantly smaller than the remaining nineteen sensors. These nineteen sensors, reflecting specifically prehensile in-hand manipulation as postulated in the introduction, produced ECs consistently within a 90% confidence interval (CI) range (0.05 < *p* < 0.95). They included all ten finger bend sensors, all four ab/adduction sensors and three pressure sensors, P1 – P3, previously shown predominantly involved in this manipulation task (8). Two sensors describing the deformation of the palm: palm arch and thumb cross, also produced significant ECs. These relevant sensors were submitted to further analysis.

A PCA of each of the six runs for each subject yielded a total of 132 analyses for each hand. The Guttman-Kaiser criteria for salient PCs yielded a mean value of 3.73 ± 0.84 for the left hand and 3.95 ± 0.82 for the right. Since the first three PCs of each run yielded mean fractions of variance explained of 81% ± 6% for the left hand and 78% ± 6% for the right, further analysis was restricted to these first three PCs.

After the realignment described in Methods, the ECs of the first three PCs of all subjects and runs, i.e. 3 × 132 = 396 sets of ECs for each hand, were assigned to one of three clusters according to K-means clustering as described above. As shown in Figs. 3, 98 PC1’s were assigned to Cluster1 of the right hand and 105 PC1’s to Cluster1 of the left. The Fisher’s exact test indicated no significant difference between hands regarding the number of assignments. The numbers of PC2’s assigned to Cluster 2 were 82 for the right hand and 102 for the left; they differ significantly at level, *p* < 0.01. Finally, as shown in Fig. S1 of Supporting Information, the numbers of PC3’s assigned to Cluster3 were 74 for the right hand and 91 for the left; they differ significantly at level, *p* < 0.05. Further comparison of Cluster1’s reveals that the means and confidence intervals of the correctly assigned PCs are comparable, but that the spread of distances for the misassigned PC1’s is markedly greater in Cluster1 of the right hand. Both the means and confidence intervals of the Cluster2’s and Cluster3’s are greater than those of the Cluster1’s for both hands. Thus, the left hand appears to show a clearer pattern of PC assignments to clusters. Regarding misassignments, 32 of 34 of the misassigned PC1’s of Cluster1 are assigned to Cluster2 for the right hand; 20 of the 27 misassigned PC1’s of Cluster1 are assigned to Cluster2 for the left hand. The difference indicates a trend: *p* < 0.07. Furthermore, 41 of 58 missassigned PC3’s of the right hand and 27 of 43 PC3’s of the left hand are assigned to their respective Cluster2’s, suggesting a mutability between the two, although the centroids are not significantly correlated; *p* < 0.14 for the right hand and *p* < 0.19 for the left.

**Fig. 4:**
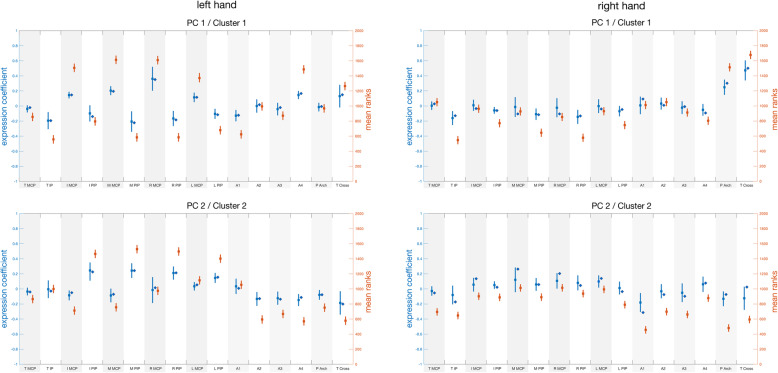
Spatial sensor patterns for PC1 and PC2 of left and right hands. The means and standard deviations of the expression coefficients determined for the dominant PCs in a cluster are represented by blue circles and bars. The adjacent diamonds denote the cluster centroids. The means and standard deviations of the ranks according to the Kruskal-Wallis analysis are represented by red circles and bars. The y-axes are coloured correspondingly; the x-axes label the sensors as in Fig. 1. A and Table 3

The mean spatial trajectories related to the end phalanges and joint sensors are shown in the animation calculated for subject ID10 (Video 1). The expression coefficient (EC) of a specific sensor expresses only the relative extent of (1) movement in the main plane of a joint: positive values indicating flexion and adduction and negative extension and abduction, or of (2) local pressure: positive values indicating less pressure and negative more pressure. In a particular principal component, the coefficient represents a phase of the trajectory. Figure 4 shows the expression of the 16 bend sensors related to the joints in the Clusters 1 and 2 for right and left hands. Complete sensor patterns for PC1, PC2 and PC3 of both hands, including the pressure sensors, are displayed in Supporting Information (Fig. S3).

An omnibus Kruskal Wallis analysis of the correctly assigned ECs indicated in red and summarized in Table 2 showed clearer inhomogeneities among the sensor patterns than the means and cluster centroids indicated in blue, at p < <e^− 10^ for PC1–3 on both sides. Post-hoc multiple comparison test using the multicompare matlab program of the ranks validated the salience of three groups of sensors: thumb cross with palm arch as well as MCP and PIP joints of the fingers exhibited highly significant variations due to phase differences between changing thumb to finger oppositions, satisfying a *p*-value of < 0.05 after correction for multiple comparisons according to Bonferroni. These main dynamics were: (1) The patterns of the Cluster1’s showing positive EC of thumb cross, prominent together with palm arch on the right, indicating marked opposition transmitted by CMC joint. In the right hand, the PIP joints and to a lesser degree the MCP joints of all fingers are out of phase (as related to varying negative ECs); in the left, the MCP joints of the fingers are in phase with the thumb CMC joint (both with positive EC) whereas the PIP joints are out of phase (negative EC). (2) The parameters of the Cluster2’s reveal in the right hand simultaneous activation of the PIP and MCP joints of the fingers (relatively positive EC); out of phase are all thumb joints (relatively negative EC). In the left hand, activation of the PIP joints of all fingers dominates (positive EC), while thumb cross (CMC joint), palm arch and MCP joints of the finger sensors are out of phase (varying negative ECs). – The interdigital sensors, measuring abduction versus adduction, indicated rather a neutral joint position according to ECs, except for A4 in Cluster1’s on the left (with positive EC consistent with adduction between ring and little finger) and for A1 in Cluster2’s on the right (with negative EC consistent with abduction between thumb and index finger).

**Table 2:**
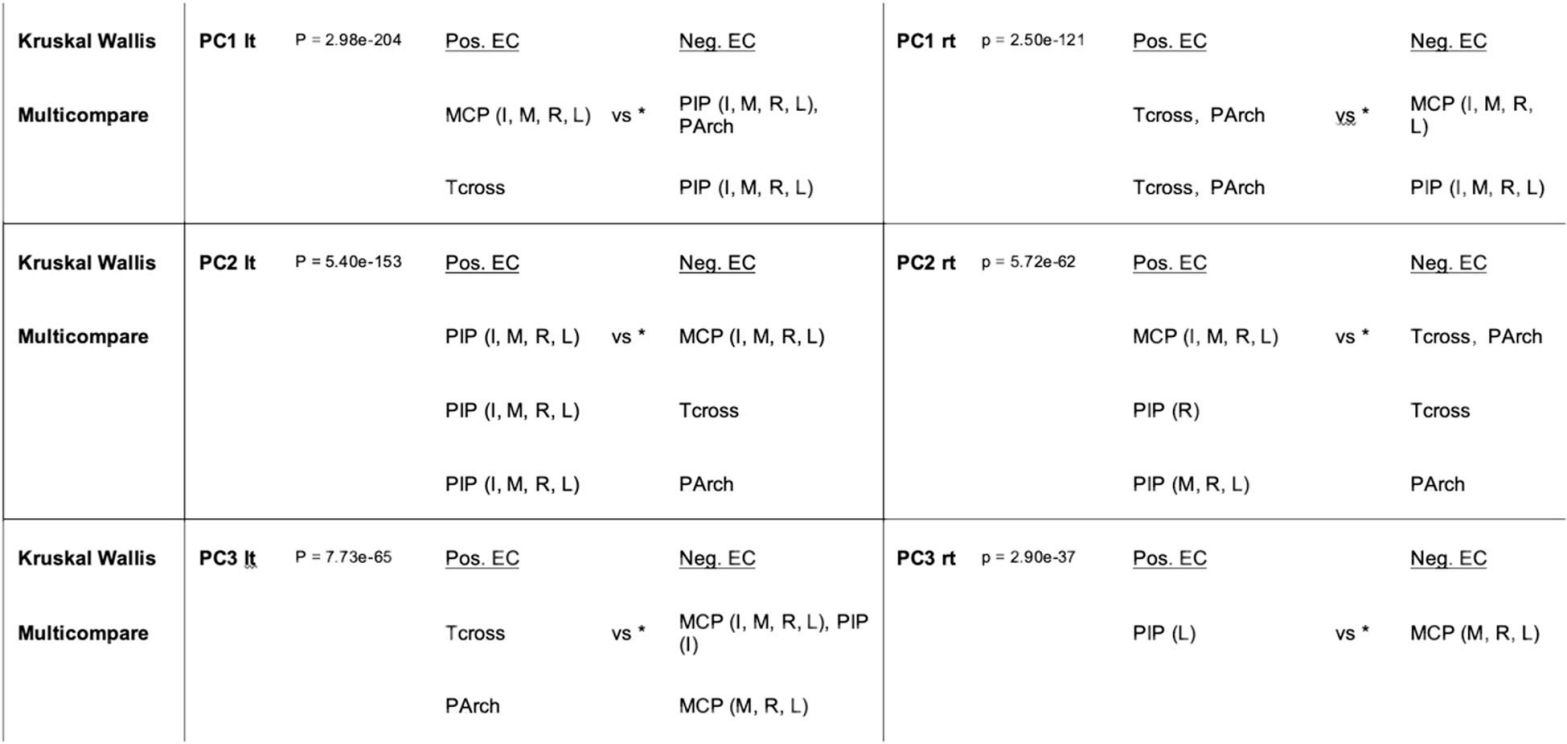
Non-parametric tests of prominent bend sensor ECs and post-hoc pairwise analysis

Kruskal Wallis and Multicompare analysis implemented in Matlab

* All shown differences for post-hoc Multicompare are significant at p < 0.05 after correction for 19 comparisons according to Bonferroni Abbreviations: PC Principal component, EC expression coefficient, Rt Right, Lt Left, MCP Metacarpo-phalangeal joints (I Index, M Middle, R Ring, L Little), PIP Proximal interphalangeal joints (I, M, R, L); Tcross, Thumb cross sensor related to carpo-metacarpal (CMC) joint of thumb; PArch, Palm Arch

According to EC the sensor patterns of Cluster3’s indicated mostly neutral joint positions on the right; on the left the thumb cross predominates indicating thumb opposition transmitted by CMC joint of thumb. Pressure sensors suggest relatively elevated pressure over the pad of the moving thumb in comparison to that over index and middle finger pad in Cluster1’s and Cluster3’s of both sides (Fig. S3).

### Temporal sensor patterns

**Fig. 5:**
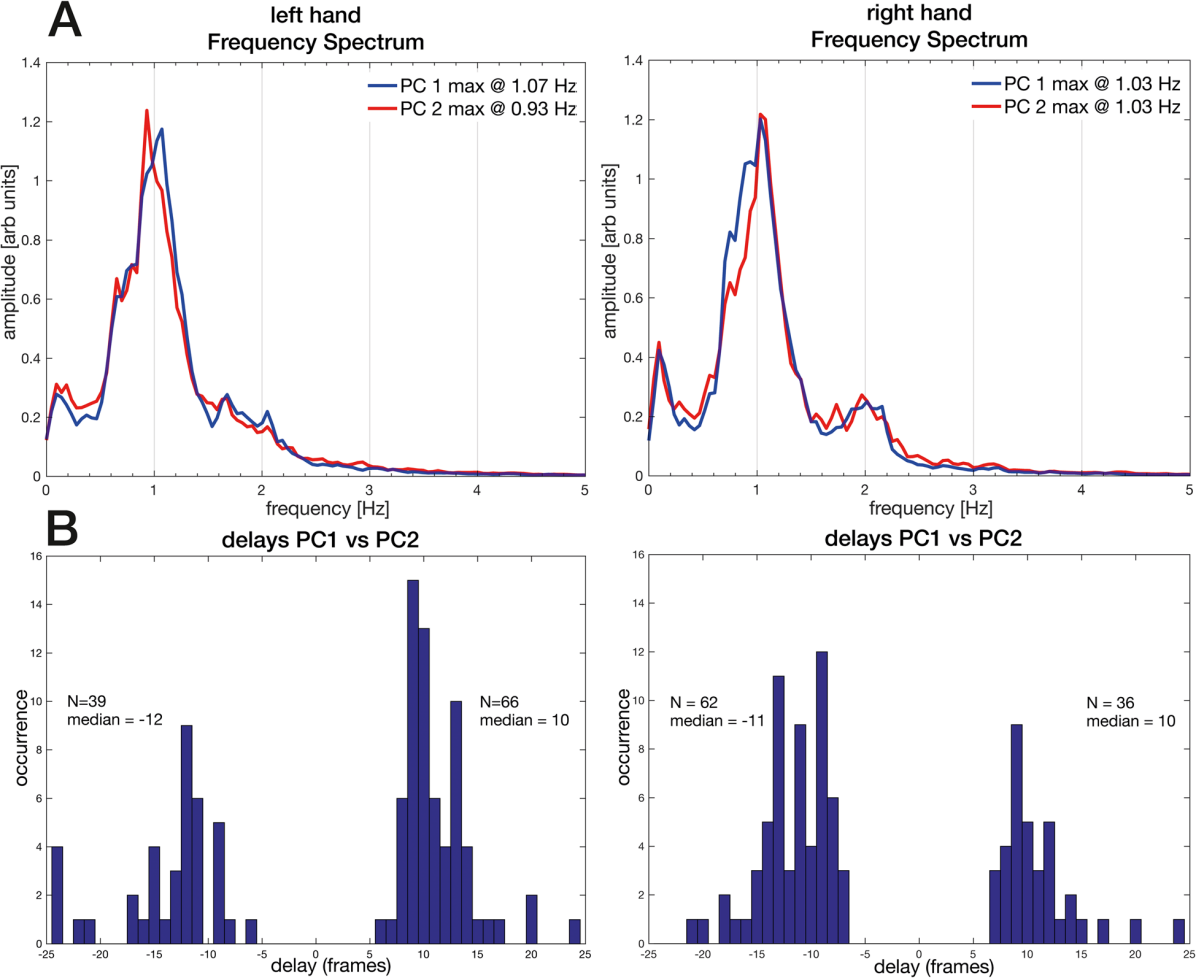
Temporal sensor frequencies for PC1 and PC2 and delays between themselves. The temporal sensor frequencies and delays fpr PC1 and PC2 of left and right hands, calculated for dominant components of a cluster. **a** Normalized frequency spectra in which blue denotes PC1 and red PC2, and **b** histograms of the delays between PC1 and PC2 in frames (1 frame = 0.02 s). Almost all the represented dominant components of PC1 and PC2 arise within 1 s manipulation corresponding to a related changing grasp configuration at that time window

We present below two types of temporal analysis in order to compare the motion of right and left hands: analyses (1) of the principal component time series and (2) of the complete time series of the three groups of finger and thumb sensors suggested by the spatial sensor patterns. The mean frequency spectra of the dominant PCs shown in Fig. 5 are very similar for the two hands. Determined mainly by the instructed task the frequency was of about 1 Hz; the spectra of PC1 showed a peak at 1.03 Hz for the right hand and at 1.07 Hz for the left. For PC2, the spectra showed peaks at 1.03 Hz and 0.93 Hz, respectively.

The negative and positive time delays between PCs shown in Fig. 5b confirm the independence observed in the spatial patterns. Almost all delays between PC1 and PC2 occur within a time window of 1 s, i.e. 50 frames: 98% for the right hand and 90% for the left. The difference is marginally significant: *p* < 0.05, two-tailed. Moreover, the asymmetries of the delay distributions differ significantly between hands at the level, *p* < 0.001. The distributions of delays between PC1 and PC3 within the same time window are broader: 74% for the right hand and 70% for the left, which are not significantly different. To facilitate comparison of all delay distributions, Fig. S4 displays them for a time window of 2 s, i.e. 100 frames.

Analysis of the complete time series of the three groups, comprising ten finger bend, palm arch and thumb cross sensors, is summarized in Table 3. It reveals no significant frequency differences between respective finger groups of the left and right hands nor among the three groups of each hand. All groups reproduce the instructed frequency within statistical deviations.

**Table 3:** Comparison of frequencies for dominant groups of finger sensors

Sensor groups	Left hand (*n* = 105 runs)		Right hand (*n* = 98 runs)		*p*-value left vs right (Mann-Whitney-U-Test)
	mean	± SD	mean	± SD	
Thumb (4 sensors, incl. Palm Arch)	1.01	0.10	1.04	0.13	0.20
MCP (4 sensors)	1.01	0.13	1.01	0.12	0.98
PIP (4 sensors)	1.01	0.11	1.02	0.13	0.17
*p*-value Friedman’s Test within one hand (thumb, MCP, PIP)	0.78		0.17		

Of the the 19 relevant sensors, the 12 most prominent sensors are grouped as Thumb (IP, MCP, Thumb Cross and Palm Arch), MCP (of fingers) and PIP (of fingers) sensors

### Graph analysis of selected sensor time series

The time series of the 12 MCP/PIP finger bend, palm arch and thumb cross sensors included in the PCA and cluster analyses resulted in a weighted, undirected network for each hand. The number of positive weights: 29 of 66 edges or 43.9%, and negative weights: 37 of 66 edges or 56.1%, was equal in both hands. The edges with the highest positive weights connect the ring MCP and little MCP joints (0.869) of the left hand and the ring PIP and little PIP joints (0.854) of the right hand. The edges with the lowest negative weights connect the thumb MCP and ring MCP joints (− 0.421) of the left hand and the thumb MCP and little MCP (− 0.503) of the right hand.

**Fig. 6:**
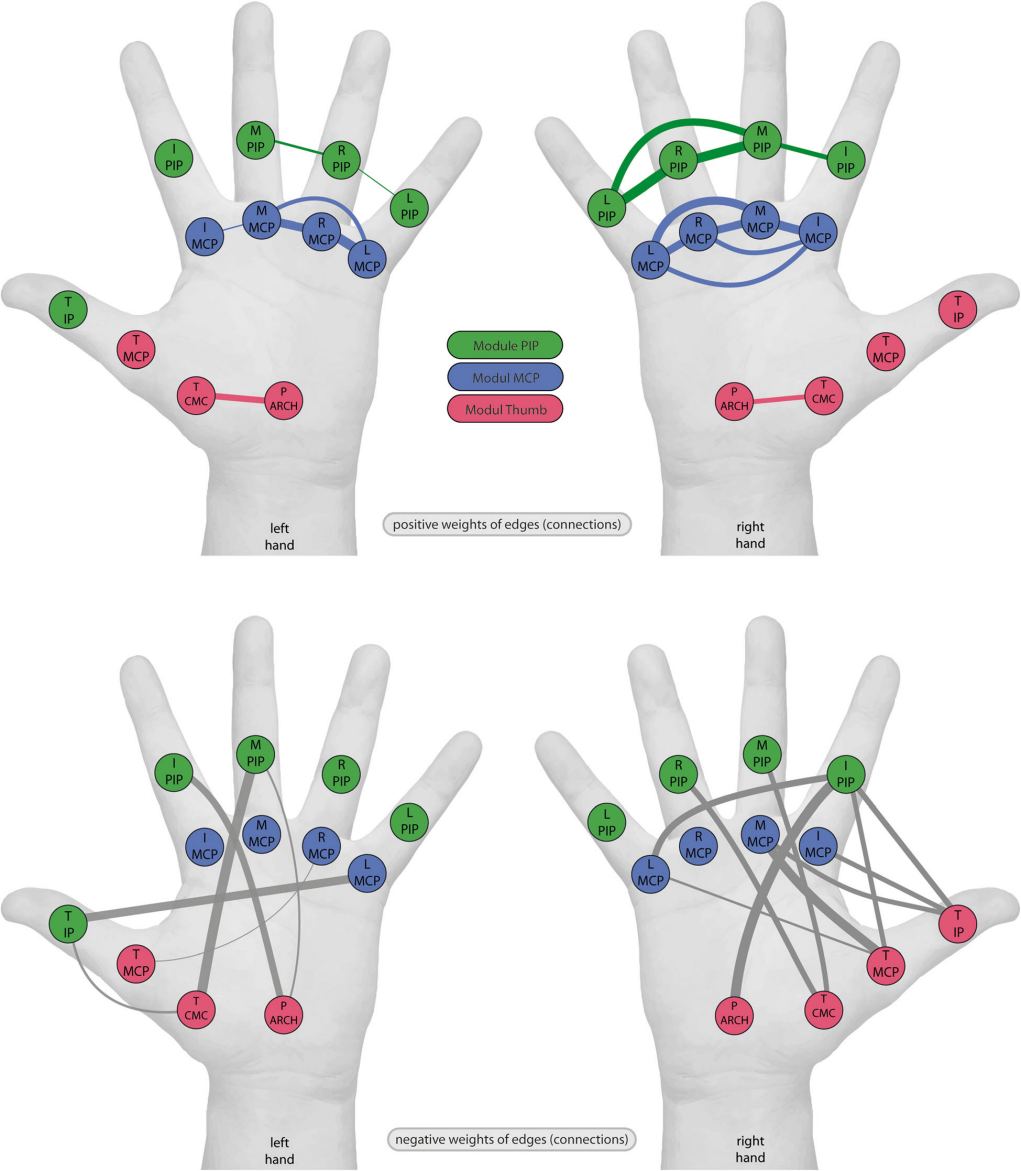
Graph networks and modular organization of finger movements. Pictorial representation of graph analysis and networks for left and right hands as related to the mainly involved joints by the task, with positive weights in the upper row and negative weights in the lower row. Nodes’ colour displays the modular structure in each hand, almost identical on the right and left. Nodes are denoted by their sensor labels, the relative weights of the connections indicated by the thickness of the lines between them (at a threshold of 0.35 in the upper row where high positive weights are represented parallel to thickness, and at a threshold of − 0.35 in the lower row where low negative weights are represented inverse to thickness). Note the strong intramodular connections in the positive weighted graph and the strong intermodular connections in the negative weighted graphs

The networks of positive weights shown in Fig. 6, thresholded at 0.35 for better illustration, reveal three strongly connected sub-networks in each hand and three modules. One network in blue connects all MCP joints and another in green all PIP joints; these joints comprise also two of the modules. A third sub-network and module in red features a strong connection between palm-arch and thumb cross. The two isolated nodes of the right hand, thumb MCP and IP, are members of the third module, whereas the thumb IP joint of the left hand is a member of the PIP module. The networks of negative weights shown in Fig. 6, thresholded at − 0.35 for better illustration, manifest the same modular structure as the positive weights, but the connections are intermodular. In the right hand, the connections between palm arch and index PIP and between thumb MCP and middle MCP are particularly strong; less strong are the connections between thumb CMC and ring and middle PIP, between thumb MCP, little MCP and index PIP, and between thumb PIP, middle and index MCP and index PIP.

Despite the differences between left- and right-hand networks suggested by Fig. 6, the global efficiencies and small world properties of the networks do not differ significantly. The mean global efficiencies of the unthresholded networks were 0.95 ± 0.03 for the left and 0.94 ± 0.02 for the right hand, indicating no significant difference: *p* < 0.11. The mean small-world propensities, φ, were 0.53 ± 0.20 for the left and 0.49 ± 0.21 for the right hand, implying no significant difference: p < 0.11. The mean small-world indices, σ, were 1.39 ± 0.59 for the left and 1.45 ± 0.55 for the right hand, implying again no significant difference: *p* < 0.12. These values indicate that both networks show small-world properties, implying substantial clustering and small path lengths.

### Temporal evolution of finger movements in space

**Fig. 7:**
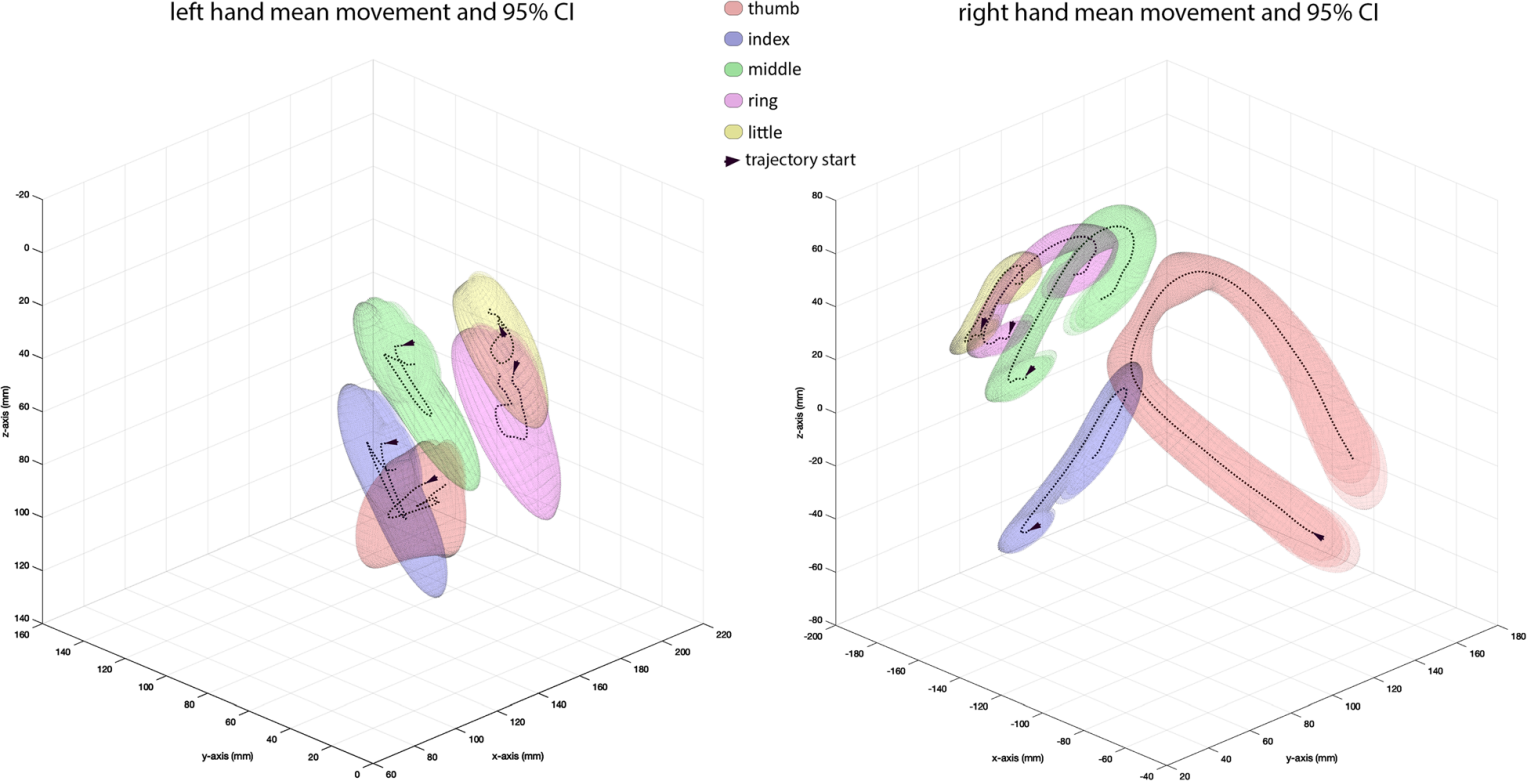
Three-dimensional representation of the finger trajectories in subject ID 10. Finger trajectories in 3 dimensions shown for left and right hands were derived from six runs of a single right-handed subject (ID 10). The black dotted lines indicate the mean position of the finger tip sensors, P1–5, and the colours the 2 s tubes of the trajectories. Note (1) the opposed position of the thumb to fingers on both sides, while the workspace is considerably restricted on the left compared to the right; and (2) the clockwise rotation of the spatial trajectories involving thumb and fingers on the right, and the anticlockwise rotation on the left

To illustrate the spatial finger trajectories recorded during the special acquisition of 3D data for subject ID10, we focused on the five sensors located at the ends of the distal phalanges, i.e. P1–5. The trajectories depicted in Fig. 7 are repeated at median frequencies of 0.78 Hz by the left hand and 0.83 Hz by the right with interquartile ranges of 0.68–0.95 Hz and 0.75–0.92 Hz, respectively. These are slightly less than the frequencies measured by the twelve sensors that dominate the spatial sensor patterns of the subject cohort. The slightly slower repetition frequencies of the left hand accompany shorter trajectories and slower finger speeds than those of the right hand. As shown in the Supporting Information (Fig. S5, Table S2), the middle finger tip of the left hand and the thumb tip of the right hand were the fastest with median speeds: 22 cm/s and 32 cm/s, respectively. The middle finger tip of the right hand showed the second fastest speed for that hand, 19 cm/s, only slightly faster than the ring finger tip, whereas the thumb tip of the left hand yielded the second slowest speed for that hand, 15 cm/s.

## Discussion

The goal of our study was a spatial and temporal description of the dynamic finger movements involved in regularly repeated tactile manipulations in right-handed healthy volunteers whose ages matched those of stroke survivors. The instruction video, immediately preceeding execution, provided spatial and temporal cues for the finger movements prior to execution, and thus supported pre-attentive sensory processes whereas execution is based on proprioception [31, 32]. In contrast to previous studies reporting finger trajectories in single reach-to-grasp tasks, in everyday activities [33–37] and in pure grasping tasks involving intrinsic hand movements [15, 38], we explored a sequence of defined motor actions typical of exploration during somatosensory discrimination in the macroscopical domain [39]. A constituent motor act of our task, manipulation of an almost regular cuboid, is shown in Fig. 1c. From a hand-centric view, the fingers interact with the object using so-called transitive movements in a workspace tightly adapted to the objects [40] as shown in Fig. 7. These movements are accompanied by motion of the object, which requires at least two fingers to hold the object while the perpendicular finger positions it [25]. During this interaction occurs a continual change of finger configurations directed to contacts at the edges and vertices of the object [4] while the fingers in contact are replaced by free fingers once they have reached joint limits of a finger pair [41]. Thus the precise handling observed is prehensile motion within the contacting hand (see taxonomy in [25]). In contrast to the hand-centric view, the object-centric view postulates that perceived attributes of the object may evoke motor acts during pure manipulation equivalent to those during active touch in object exploration, i.e. stresses the aspect of the hand as sense organ [37]. An analysis of natural hand movements confirmed the similarity of finger joint trajectories in both classes of prehensile in-hand activity [38].

Derived from the 19-dimensional glove sensor space, the first three PCs of each run explained 75 to 80% of the variance, and were thus salient according to the Gutmann Kaiser criterion [26]. This low dimensionality is consistent with the observations of Belic and Faisal [34], Jarassé et al. [36] and Ingram et al. [42] in tasks involving motor control of daily reach-to-grasp activities, of bilateral hand movements and of natural, spontaneously generated hand movements, respectively. The first principal component for both hands accounted typically for one half or more of the variance explained by the salient components. K-mean clustering permitted a comprehensive analysis of the subject cohort with respect to homogeneity of sensor pattern ECs. The number of PC1’s assigned to corresponding Cluster1’s was comparable for both hands. The other two PCs showed significant differences between hands, as indicated in Figs. 3 and S1 of the Supporting Information. A greater number of PCs were correctly assigned to the corresponding clusters for the left hand as compared to the right. Moreover, the majority misassigned PC2’s in Cluster 2 were assigned to Cluster 3 and vice versa. These observations suggest a more flexible handling strategy of the right hand. In the context of stochastic optimal feedback control proposed by Todorov and Jordan [43], the mutable PCs might represent variability in task-irrelevant dimensions between motor acts, and reflect fluent action in the dynamic activity of the right hand without exceeding normal limits. If *task-irrelevant* is substituted for *salient*, these observations are consistent with the observations of Faisal et al. [44], who found in archaeological toolmaking a correlation between the complexity of an underlying hand motor task and the number of salient components.

Represented in Fig. 4, the spatial patterns of single motor acts exhibited by the salient PCs appear to be encoded mainly by twelve of the nineteen sensors. These twelve imply three groups of coordinated and synergistic finger movements: a 1st group related to the carpo-metacarpal (CMC) joint of the thumb together with palm arch sensor; a 2nd group related to the metacarpophalangeal (MCP) joints of the fingers; and a 3rd group related to the proximal interphalangeal (PIP) joints of the fingers. Trajectories associated with these joints have been shown to be stereotypical and characterized by multicollinearity of the MCP and PIP joints [45]. The Kruskal Wallis nonparametric analysis of the EC distributions established varying interactions among thumb and fingers as expressed by their mutual asychrony, i.e. the opposition of the CMC (carpo-metacarpal) joint of the thumb and flexion of MCP and PIP joints, during the phases of the task performance represented by the principal components. These phases consist presumably of different grasp configurations composing the motor act demanded by the task. The asymmetric expression in PC1 of the CMC joint of the thumb together with palm arch on the right and the MCP joint of the three middle fingers on the left is noteworthy. It marks dynamism and synchrony between thumb and shaping the hand on the right [23, 46], and rather stabilizing a holding function on the left. Regarding the time series associated with the salient PCs, the frequency spectra shown in Fig. 5 evidence a clear peak at 1 Hz, the frequency of the repeated cuboid manipulations shown in the video immediately prior to execution of the task. The time delays between the dominant and subdominant PCs for both hands confirm their independence. They are of both signs, but are significantly asymmetric with the dominant sign differing according to hand. Their common task frequency and short delays within a 1 s time window, corresponding to one motor act, confirms finger gaiting as principal mechanism underlying one motor act, which prevents loss of the cuboid [13]. This is the first time that finger gaiting is observed in a human sensori-motor task fundamental to the haptic exploration of objects, e.g. for shape perception, during which the fingers hold the object while it is surveyed by the thumb [39, 47].

The time series of the 12 sensors comprising three groups posited to engage in synergistic movements of the MCP and PIP joints of the fingers, and the thumb joints together with palm arch also yielded median frequencies of 1 Hz in both hands. As suggested in Table 3, the frequencies of the fingers are consistent and homogenous for the three groups in each hand, suggesting an intrinsic harmonic, synchronous organization (cf. [46, 48]). Complementary analysis of the time series of the 12 sensors using graph theory establishes the modular organization underlying this multifinger task (Finger 5), supplementing earlier assumption of modular organization of finger movements relying on suprathreshold magnetic stimulation of the human motor cortex [49]. It shows for the right hand positive correlations among analogous joints, MCP and PIP, of four fingers and between palm arch and thumb cross, and negative correlations among joints of palm arch and thumb and a majority of the finger joints. The left hand shows similar, but fewer connections. The connections of the positively correlated nodes at the MCP and PIP joints may reflect repeated synchronous motor acts during the task and encode time varying motor information essential for a dynamical system engaged in manipulation [50]. The dense interconnections between MCP and PIP joints of adjacent fingers confirm the positive correlations between these joint pairs posited in the spatial patterns [48]. The connections of the negatively correlated nodes reflect anticorrelation between thumb and PIP and to a lesser degree MCP joints, compatible with their asynchrony among each other in the motor act patterns shown in Fig. 4. The graph analysis indicates high local movement efficiency and short paths among the interconnected joints, corroborating temporal organization during the task within and among joint groups as detailed above [46, 51, 52]. The graphs of both hands exhibit small world characteristics, i.e. specifically high global efficiency as a measure of information exchange among subnetworks. Thus, the capacity for functional parallel synergy within the modules is equally great in both hands [53, 54].

The spatial finger trajectories shown in Fig. 7 illustrate for a single subject the temporal evolution of the finger tips in space. They represent the tangential sliding of the fingers as they encompass the cuboid. The paths are restricted, comprising only a small percentage of available workspace and limited degrees of freedom [55]. The workspace occupied by the trajectories of the right hand is much greater than that of the left hand, suggesting the greater variability associated with optimal feedback control [43] posited in the spatial patterns. The longer trajectories of the right hand imply that the speeds of the finger tips are greater [56], since the repetition frequencies are subordinated to the manipulation frequency of 1 Hz. The manifest differences between right and left hands observed in the spatial and temporal patterns of the PCA, in the graph analysis and in the trajectories of Fig. 7 may reflect the distinct roles of left and right hands in everyday human activities as reported in studies of bimanual tasks. In these tasks, the left hand provides rather stable postural support while the right assumes a more dynamic, spatially extended role [44, 57].

Limitations are inherent in the choice of object to be explored and in precise instructions of how it should be explored. We relied deliberately on a theoretical model of human somatosensory exploration of kinesthesia developed and validated by Roland and Mortensen [58] in which information is sampled successively and sequentially. Hence, the application of this well-studied task allows generalization specifically to recognition of macroscopical aspects of objects, e.g. shape [22]. Moreover, multiple precision grips of the involved fingers during a sequence of consecutive manipulations are subject to failure above a grasping frequency threshold of 2 Hz. The selection of a cuboid as object and exploration frequency of 1 Hz was made to provide a prototypical task for the study of the post-stroke recovery of coordinated hand motor skills in a clinical context and of significance for daily motor needs cf. [54].

## Conclusions and practical implications

Using a digital data glove, we have exposed new spatial and temporal aspects of the object manipulation underlying tactile exploration. A comprehensive data analysis has revealed: 1. A hierarchy of three elementary grasp configurations revealed using PCA. Occurring at the prescribed frequency of 1 Hz with distinct delays between configurations within the 1 s time window, and thus constituting one complex motor act, these configurations represent finger gaiting. 2. A functional network of high global efficiency revealed by graph analysis of the time series of the twelve finger and thumb sensors most involved in the configurations. The network could be partitioned into three modules consisting of a. MCP and b. PIP joints of the fingers and c. the thumb joint and palm arch, and reflecting intramodular synchrony and intermodular asynchrony. 3. Striking lateral differences confirmed in the 3D reconstruction of the manipulations in a single subject. The right hand exhibited a larger workspace of opposed thumb and fingers than the left hand, confirming the greater variability of spatial motor patterns proposed in the cluster analysis of cohort principal components. In addition to providing a prototypical task for the study of the post-stroke recovery, the sequence of basic manipulations required by the task might serve as a model of human TOR involving prehensile in-hand manipulation relevant also to the development of robotic tactile perception systems [5, 59, 60]. Concerning post-stroke recovery, this study offers a standard for monitoring sensori-motor defects. The practical importance resides in the observation that preserved partial dexterity after stroke, including motor control during active touch, has been shown to be an important resource in rehabilitation of the upper extremity [1, 2]. Moreover, post-stroke motor function may depend heavily on the recovery of sensory function [22, 61], and persistent somatosensory impairment may be associated with slow recovery and persistent dependency [62–64]. Possible use of the task in the future might be integration of the instruction video into a rehabilitation program supplemented by visual feedback of training sessions. In the future a more ambitious application might be the addition of vibro-tactile stimulation at the site of the glove’s bend sensors which, mediated by robot-assisted proprioceptive feedback, could progressively facilitate motor performance [65, 66].

## Supplementary information

**Additional file 2: Figure S1.** Fraction of variance for PC1, PC2 and PC3. Fraction of variance related to PC1, PC2 and PC3 is related to each subject and run. A negative correlation of the fraction of variance in PC1 with the number of significant PCs according to the Guttman-Kaiser criteria was found in the left (Pearson’s r = − 0,60, *p* < 0.05) and right (Pearson’s r = − 0.59, *p* < 0.05) hand, which highlights PC1 as a dominant pattern. **Figure S2.** K-mean cluster classification of spatial sensor patterns for PC3 of left and right hands. K-mean cluster classification of spatial sensor patterns for PC3 of left and right hands is shown where green dots are runs assigned to PC3/Cluster3. The color blue donates PC1s and red PC2s. The distances are derived from the correlation between the cluster centroid and the spatial pattern of a run. The mean distances and two standard deviations of the dominant PCs of a cluster are represented as solid and dotted lines. The median distances are represented by the solid lines. The number of PC3 assigned to Cluster 3 were 74 for the right and 91 for the left hand (significant lower frequency related to the right hand according to Fisher exact test, p < 0.05). **Figure S3.** Spatial sensor patterns for PC1, PC2 and PC3 of left and right hands. The means and standard deviations of the expression coefficients determined for the dominant PCs in a cluster are represented by blue circles and bars. The adjacent diamonds denote the cluster centroids. The means and standard deviations of the ranks according to the Kruskal-Wallis analysis are represented by red circles and bars. The y-axes are coloured correspondingly; the x-axes label the sensors as in Fig. 1. Figure S3A and S3B represent the patterns of PC1 and PC2 of left and right hand, here including additionally the pressure sensors P1-P3 which has been mentioned and discussed in the main body of the paper. Figure S3C shows PC3, exhibiting a reduced signal to noise ratio in comparison to PC1 and PC2. In comparison to Fig. 3, Figure S3 is extended with the three pressure sensors involved in the task (the pressure sensor of the thumb (P1), the pressure sensor of the index (P2), and the pressure sensor of the middle finger (P3)). Note: weakly developed pattern on the left similar to that of cluster1_R, significant differences restricted to a few MCP joints versus PIP joint of the little finger on the right. MCP, Metacarpo-phalangeal joints (T thumb, I, Index; M, Middle; R, Ring; L, Little); PIP, Proximal interphalangeal joints (T, I, M, R, L); Tcross, Thumb cross sensor related to carpo-metacarpal joint of thumb; PArch, Palm Arch; P1, P2, P3 pressure sensors related to thumb (P1), index (P2) and middle finger (P3); A1, adduction sensor between thumb and index finger; A2, adduction sensor between index and middle finger; A3, adduction sensor between middle and ring finger; A4 adduction sensor between ring and little finger. **Figure S4.** Temporal sensor frequencies and delays for PC1 and PC3 of left and right hands. Temporal sensor frequencies for PC1 and PC3 of left and right hands and delays for PC1 to PC2 as well as for PC1 to PC3 are shown, calculated for dominant components of a cluster. Quality control using a 2 s time window while one manipulation occurs at 1 Hz. A) Normalized frequency spectra in which blue denotes PC1 and green PC3 (upper row), and B) histograms of the delays between PC1 and PC2 (middle row), and PC1 and PC3 (lower row) in frames (1 frame = 0.02 s). Almost all the represented dominant components of PC1 and PC 2 and to a lesser degree PC3 (between 70 and 74%) arise within 1 s manipulation corresponding to a related changing grasp configuration at that time window. **Table S1.** Sensimotor cohort data. **Table S2.** Finger tips’ mean speed of subject 10.

**Additional file 3: Calibration steps.** Calibration of the data glove.

## Data Availability

The datasets generated during the current study and study protocol are available in the Open Science Framework (OSF) repository at https://osf.io/jp825/ .

## Abbreviations

2PD: Two-point discrimination
CI: Confidence interval
CL: Confidence level
CMC: Carpo-metacarpal joint
EC: Expression coefficient
ECs: Expression coefficients
FIR: Finite impulse response
fMRI: Functional magnetic resonance imaging
GK: Guttman-Kaiser criteria
IP: Interphalangeal joint
JTT: Jebsen-Taylor Test
KEK: Kantonale Ethikkommission Bern
LQ: Laterality quotient
MCP: Metacarpo-phalangeal joint
MRI: Magnetic resonance imaging
MMSE: Mini-Mental State Examination
PArch: Palm Arch sensor
PC: Principal component
PCA: Principal component analysis
PIP: Proximal interphalangeal joint
PSO: Picking Small Objects
Tcross: Thumb cross sensor
TOR: Tactile object recognition
