# Supplementary material for: Sensing form - finger gaiting as key to tactile object exploration - a data glove analysis of a prototypical daily task
Source: J Neuroeng Rehabil. 2020 Oct 8;17:133. doi: 10.1186/s12984-020-00755-6 (PMC7542978; doi:10.1186/s12984-020-00755-6)
Supplement: Supplementary file 2 — Additional file 2: Figure S1. Fraction of variance for PC1, PC2 and PC3. Fraction of variance related to PC1, PC2 and PC3 is related to each subject and run. A negative correlation of the fraction of variance in PC1 with the number of significant PCs according to the Guttman-Kaiser criteria was found in the left (Pearson’s r = − 0,60, p < 0.05) and right (Pearson’s r = − 0.59, p < 0.05) hand, which highlights PC1 as a dominant pattern. Figure S2. K-mean cluster classification of spatial sensor patterns for PC3 of left and right hands. K-mean cluster classification of spatial sensor patterns for PC3 of left and right hands is shown where green dots are runs assigned to PC3/Cluster3. The color blue donates PC1s and red PC2s. The distances are derived from the correlation between the cluster centroid and the spatial pattern of a run. The mean distances and two standard deviations of the dominant PCs of a cluster are represented as solid and dotted lines. The median distances are represented by the solid lines. The number of PC3 assigned to Cluster 3 were 74 for the right and 91 for the left hand (significant lower frequency related to the right hand according to Fisher exact test, p < 0.05). Figure S3. Spatial sensor patterns for PC1, PC2 and PC3 of left and right hands. The means and standard deviations of the expression coefficients determined for the dominant PCs in a cluster are represented by blue circles and bars. The adjacent diamonds denote the cluster centroids. The means and standard deviations of the ranks according to the Kruskal-Wallis analysis are represented by red circles and bars. The y-axes are coloured correspondingly; the x-axes label the sensors as in Fig. 1. Figure S3A and S3B represent the patterns of PC1 and PC2 of left and right hand, here including additionally the pressure sensors P1-P3 which has been mentioned and discussed in the main body of the paper. Figure S3C shows PC3, exhibiting a reduced signal to noise ratio in comparison to PC1 an [file 12984_2020_755_MOESM2_ESM.docx]

**Supporting Information (File 2)**

**Sensing form - finger gaiting as key to tactile object exploration - A data glove analysis of a prototypical daily task**

Werner Krammer­^1,2^, John H. Missimer^3^, Simon Habegger^1^, Manuela Pastore-Wapp^1^, Roland Wiest^1^, Bruno J. Weder^1^

1. Support Center for Advanced Imaging (SCAN), Department of Diagnostic and Interventional Neuroradiology, Inselspital, Bern, University Hospital, Bern, Switzerland
2. Department of Neurology, Kantonsspital St. Gallen, St. Gallen, Switzerland.
3. Paul Scherrer Institute, PSI, Laboratory of Biomolecular Research, Villigen, Switzerland.

## **Index:**

[Figure S1. Fraction of variance for PC1, PC2 and PC3 2](#_Toc51255904)

[Figure S2. K-mean cluster classification of spatial sensor patterns for PC3 of left and right hands 3](#_Toc51255905)

[Figure S3. Spatial sensor patterns for PC1, PC2 and PC3 of left and right hands 4](#_Toc51255906)

[Figure S4. Temporal sensor frequencies and delays for PC1 and PC3 of left and right hands 6](#_Toc51255907)

[Table S1. Sensimotor cohort data 8](#_Toc51255908)

[Table S2. Finger tips’ mean speed of subject 10 9](#_Toc51255909)

**
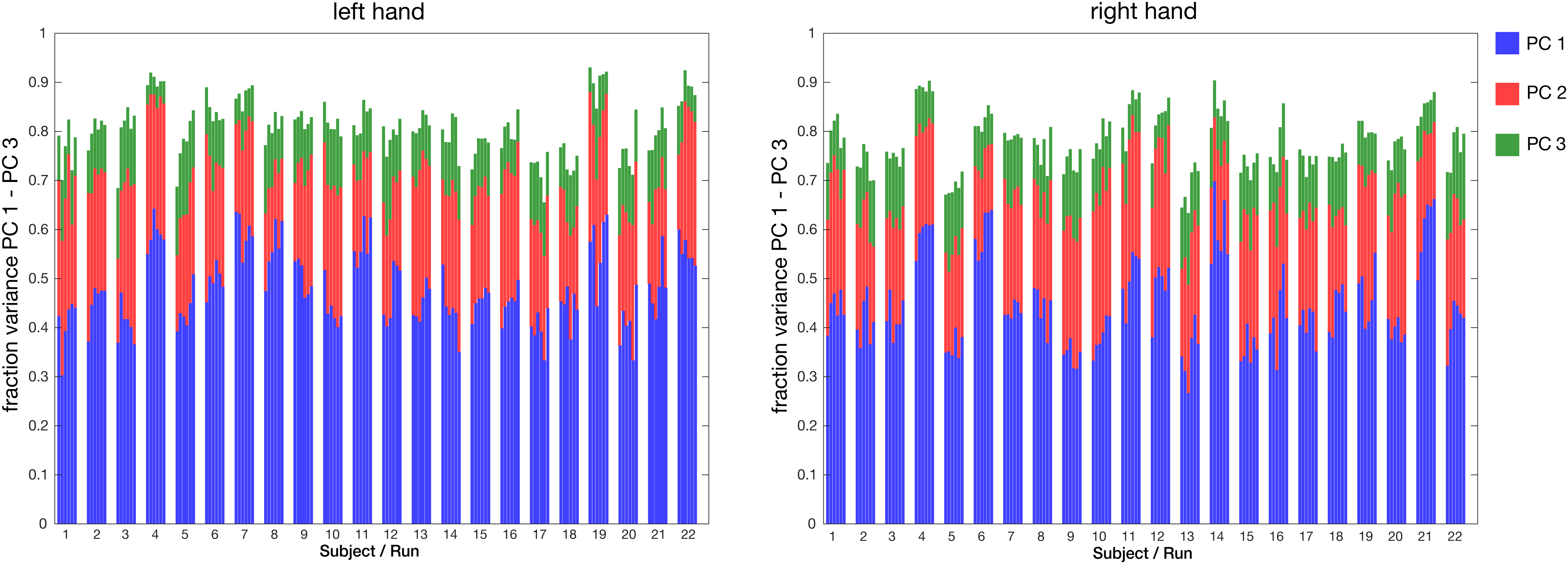
**

# Figure S1. Fraction of variance for PC1, PC2 and PC3

Fraction of variance related to PC1, PC2 and PC3 is related to each subject and run. A negative correlation of the fraction of variance in PC1 with the number of significant PCs according to the Guttman-Kaiser criteria was found in the left (Pearson’s r = -0,60, p <0.05) and right (Pearson’s r = -0.59, p<0.05) hand, which highlights PC1 as a dominant pattern.

# Figure S2. K-mean cluster classification of spatial sensor patterns for PC3 of left and right hands

K-mean cluster classification of spatial sensor patterns for PC3 of left and right hands is shown where green dots are runs assigned to
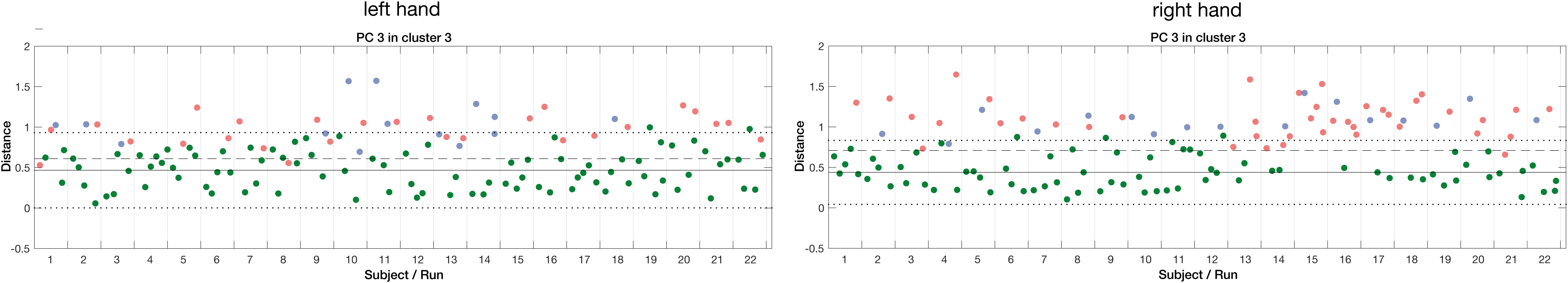
PC3/Cluster3. The color blue donates PC1s and red PC2s. The distances are derived from the correlation between the cluster centroid and the spatial pattern of a run. The mean distances and two standard deviations of the dominant PCs of a cluster are represented as solid and dotted lines. The median distances are represented by the solid lines. The number of PC3 assigned to Cluster 3 were 74 for the right and 91 for the left hand (significant lower frequency related to the right hand according to Fisher exact test, p<0.05).

**
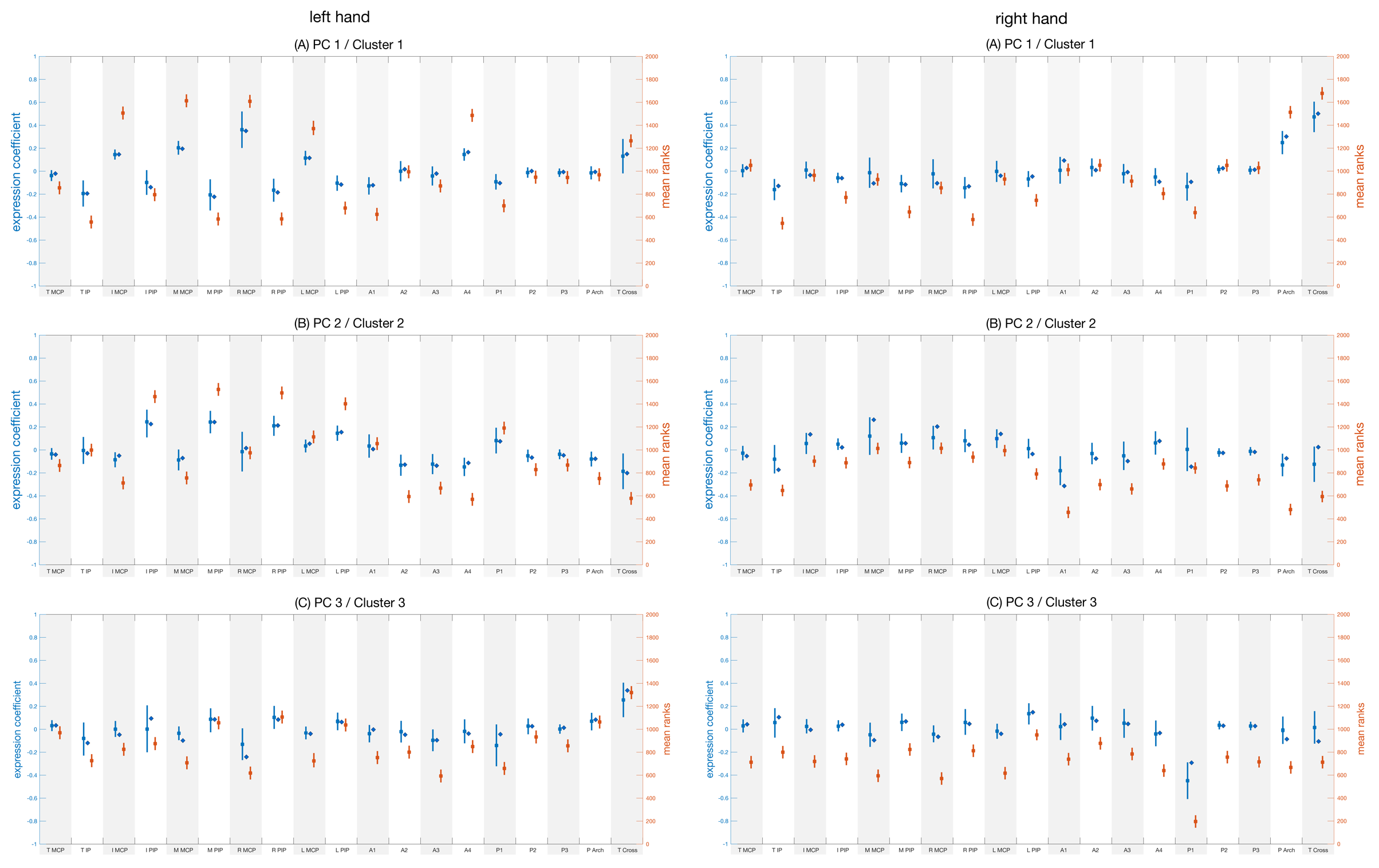
**

# Figure S3. Spatial sensor patterns for PC1, PC2 and PC3 of left and right hands

The means and standard deviations of the expression coefficients determined for the dominant PCs in a cluster are represented by blue circles and bars. The adjacent diamonds denote the cluster centroids. The means and standard deviations of the ranks according to the Kruskal-Wallis analysis are represented by red circles and bars. The y-axes are coloured correspondingly; the x-axes label the sensors as in Figure 1.

Figure S3A and S3B represent the patterns of PC1 and PC2 of left and right hand, here including additionally the pressure sensors P1-P3 which has been mentioned and discussed in the main body of the paper. Figure S3C shows PC3, exhibiting a reduced signal to noise ratio in comparison to PC1 and PC2.

In comparison to Figure 3, Figure S3 is extended with the three pressure sensors involved in the task (the pressure sensor of the thumb (P1), the pressure sensor of the index (P2), and the pressure sensor of the middle finger (P3)). Note: weakly developed pattern on the left similar to that of cluster1_R, significant differences restricted to a few MCP joints versus PIP joint of the little finger on the right.

MCP, Metacarpo-phalangeal joints (T thumb, I, Index; M, Middle; R, Ring; L, Little); PIP, Proximal interphalangeal joints (T, I, M, R, L); Tcross, Thumb cross sensor related to carpo-metacarpal joint of thumb; PArch, Palm Arch; P1, P2, P3 pressure sensors related to thumb (P1), index (P2) and middle finger (P3); A1, adduction sensor between thumb and index finger; A2, adduction sensor between index and middle finger; A3, adduction sensor between middle and ring finger; A4 adduction sensor between ring and little finger.


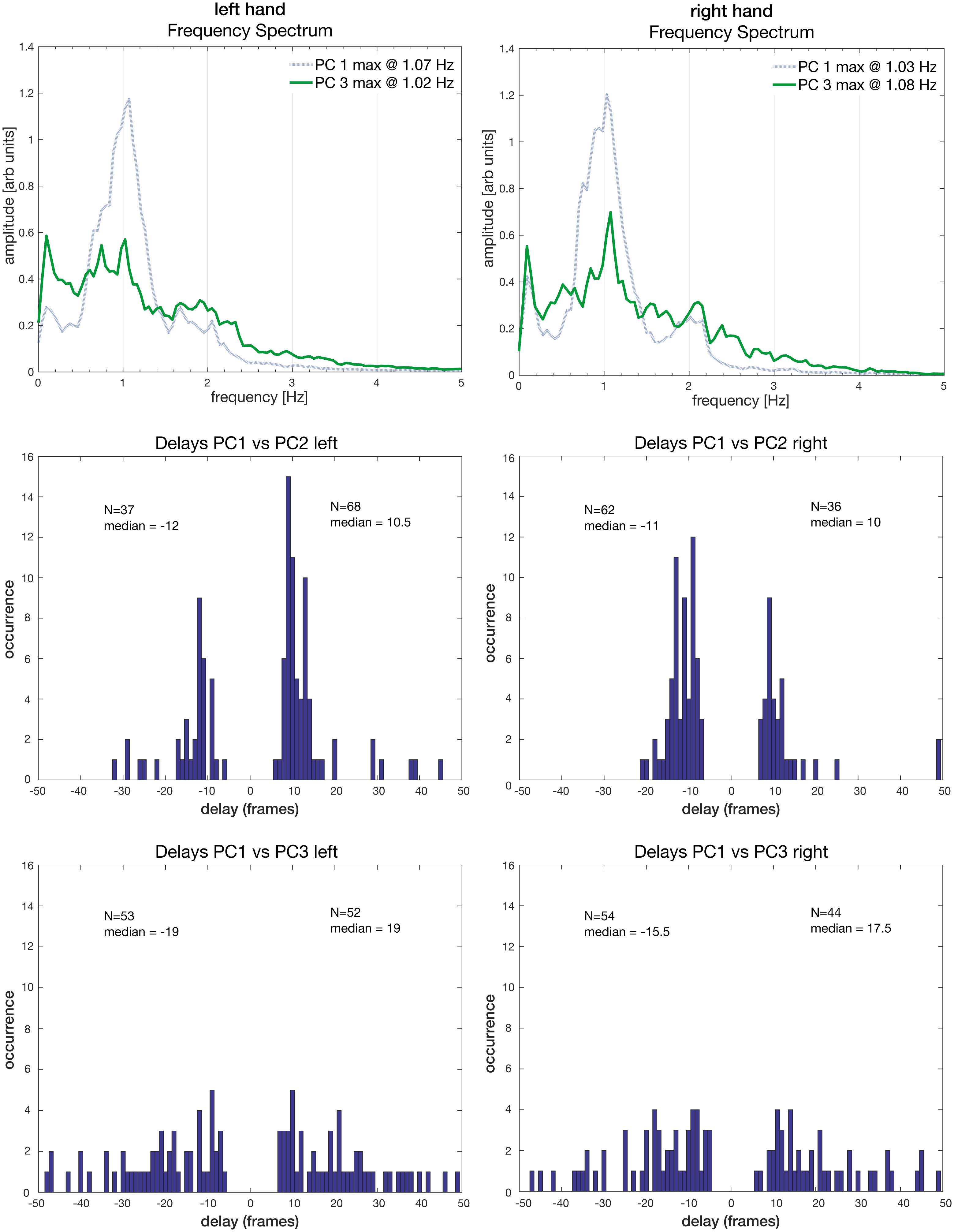


# Figure S4. Temporal sensor frequencies and delays for PC1 and PC3 of left and right hands

Temporal sensor frequencies for PC1 and PC3 of left and right hands and delays for PC1 to PC2 as well as for PC1 to PC3 are shown, calculated for dominant components of a cluster. Quality control using a 2 second time window while one manipulation occurs at 1 Hz. A) Normalized frequency spectra in which blue denotes PC1 and green PC3 (upper row), and B) histograms of the delays between PC1 and PC2 (middle row), and PC1 and PC3 (lower row) in frames (1 frame = 0.02 sec). Almost all the represented dominant components of PC1 and PC 2 and to a lesser degree PC3 (between 70 and 74 %) arise within 1 second manipulation corresponding to a related changing grasp configuration at that time window.

# Table S1. Sensimotor cohort data

|  | left hand | | right hand | | p-value  left vs right  (Mann-Whitney-U-Test) |
| --- | --- | --- | --- | --- | --- |
| Sensori-motor-parameters | median | IQR (25-75) | median | IQR (25-75) |  |
| power grip (kg) | 29 | 25.8 – 35 | 32 | 28.5 – 37.5 | 0.2621 |
| *power grip (z-score) woman** | 1.10 | 0.12 – 1.54 | 0.88 | 0.22 – 1.31 | 0.3107 |
| *power grip (z-score) man** | 0.24 | -0.39 – 1.54 | -0.03 | -0.93 – 1.91 | 0.6230 |
| precision grip (kg) | 7.5 | 6.3 – 9.5 | 8.5 | 7.5 – 10.5 | 0.2055 |
| *precision grip (z-score) woman** | -0.15 | -0.59 – 0.73 | 0.38 | -0.12 – 0.63 | 0.3978 |
| *precision grip (z-score) man** | 0.02 | -0.92 – 0.89 | -0.04 | -0.4 – 0.72 | 0.8201 |
| PSO | 6.3 | 5.4 – 7.2 | 6.4 | 5.6 – 7.4 | 0.6724 |
| *PSO (z-score) women*** | -0.58 | -1.15 – 0.18 | -0.91 | -1.53 – 0.60 | 0.5441 |
| *PSO (z-score) man*** | 0.75 | 0.27 – 1.71 | 0.34 | -0.2 – 1.45 | 0.7337 |
| two point discrimination (mm) | 4 | 3 – 4 | 3 | 3 – 4 | 0.4418 |
| *two point discrimination (z-score) woman**** | -0.93 | -1.38 – -0.49 | -1.47 | -1.47 – -0.63 | 0.0264 |
| *two point discrimination (z-score) man**** | -0.37 | -0.94 – -0.37 | -0.63 | -0.95 – -0.30 | 0.2563 |
| TOR (no. objects) | 28 | 27.3 - 29.8 | 29 | 28.3 – 30 | 0.1413 |
| *TOR (no. objects, z-score) woman**** | -0.04 | -0.64 – 0.55 | -1.17 | -1.58 – 0.47 | 0.1699 |
| *TOR (no. objects, z-score) man**** | -1.12 | -1.94 – -0.50 | -0.16 | -0.70 – 0.38 | 0.0245 |

* ­­in comparison with Mathiowetz et al. 1985

** in comparison with Jebsen et al 1969

*** in comparison with Abela et al 2019

(PSO, Picking Small Objects; TOR, Tactile object recognition; IQR, interquartile range)

# Table S2. Finger tips’ mean speed of subject 10

(n = number of analysed manipulations)

| finger | Left hand (n=93) | | Right hand (n=96) | | p-value, right vs left  Mann-Whitney-U-Test |
| --- | --- | --- | --- | --- | --- |
|  | Mean (cm/s) | ±SD | mean (cm/s) | ±SD |  |
| thumb | 15.39 | 2.66 | **32.69** | 6.20 | < 0.000 |
| index | **11.49** | 4.62 | 9.11 | 4.48 | < 0.000 |
| middle | **22.82** | 5.26 | 19.87 | 6.23 | < 0.000 |
| ring | 15.95 | 8.52 | **18.82** | 5.59 | 0.001 |
| little | **16.08** | 6.19 | 9.64 | 4.50 | < 0.000 |
