# Supplementary material for: Sensing form - finger gaiting as key to tactile object exploration - a data glove analysis of a prototypical daily task
Source: J Neuroeng Rehabil. 2020 Oct 8;17:133. doi: 10.1186/s12984-020-00755-6 (PMC7542978; doi:10.1186/s12984-020-00755-6)
Supplement: Supplementary file 3 — Additional file 3: Calibration steps. Calibration of the data glove. [file 12984_2020_755_MOESM3_ESM.docx]

**Supporting Information (Additional file 3)**

**Sensing form - finger gaiting as key to tactile object exploration – A data glove analysis of a prototypical daily task**

Werner Krammer­^1,2^, John H. Missimer^3^, Simon Habegger^1^, Manuela Pastore-Wapp^1^, Roland Wiest^1^, Bruno J. Weder^1^

1. Support Center for Advanced Imaging (SCAN), Department of Diagnostic and Interventional Neuroradiology, Inselspital, Bern, University Hospital, Bern, Switzerland
2. Department of Neurology, Kantonsspital St. Gallen, St. Gallen, Switzerland.
3. Paul Scherrer Institute, PSI, Laboratory of Biomolecular Research, Villigen, Switzerland.

# Calibration steps. Calibration of the data glove

The calibration consisted of seven calibration stages set as default program. Each calibration step has a duration of 8 seconds.

| Step 1 | maximal simultaneous flexion and extension of all fingers and thumb at a frequency of 1Hz |
| --- | --- |
| Step 2 | alternating maximum adduction and abduction of all fingers at a frequency of 1 Hz |
| Step 3 | maximal transaxial extension of the thumb (including an associated inward rotation) related to CMC joint to the little finger, and maximal flexion of MCP and PIP joints in all fingers (respectively IP joint of the thumb) |
| Step 4 | one opposing movement of the thumb to the index finger forming an “O” with medium pressure on the finger tips |
| Step 5 | same procedure as step 4 with thumb to the middle finger |
| Step 6 | same procedure as step 4 with thumb to the ring finger |
| Step 7 | same procedure as step 4 with thumb to the little finger |
